# The Application of Flexible Graphene Field-Effect Transistor Sensors in Multidimensional Biosensing and Precision Medicine

**DOI:** 10.3390/ma19132829

**Published:** 2026-07-02

**Authors:** Ting Liu, Gaozhe Cai, Qinglong Yan, Feng Shen, Jianlong Zhao, Lihua Wang

**Affiliations:** 1 School of Microelectronics, Shanghai University, Shanghai 200444, China; 2 Institute of Materiobiology, College of Sciences, Shanghai University, Shanghai 200444, China; 3 Jiaxing Key Laboratory of Biosemiconductors (A), Xiangfu Laboratory, Jiaxing 314102, China; 4 Shanghai Frontier Innovation Research Institute, Shanghai 201108, China; 5 Yangtze Delta Region Institute of Tsinghua University, Zhejiang, Jiaxing 314000, China

**Keywords:** graphene field-effect transistor, flexible sensor, multidimensional biosensing, biosensors

## Abstract

**Highlights:**

**What are the main findings?**

- The signal conversion mechanism of FGr-FETs for multidimensional biosensing is systematically summarized.
- Strategies to improve the sensing performance of FGr-FETs are summarized, including material modification, structural optimization, and signal amplification.
- A method for precise physiological signal extraction using AI-assisted graphene FET sensors is explored.

**What are the implications of the main findings?**

- Provides theoretical basis for next-gen precision medicine platforms using flexible electronics.
- Guides design of high-performance FGr-FET sensors for complex physiological environments.
- Connects flexible sensing and artificial intelligence, advancing intelligent multidimensional biosensing applications.

**Abstract:**

Flexible graphene field-effect transistors (FGr-FETs) possess ultra-high detection sensitivity, excellent mechanical flexibility, and extensive interface signal transduction mechanisms, driving the innovative development of flexible electronics. However, the accurate extraction of signals in complex physiological scenarios remains a key factor limiting the advancement of FGr-FET. This review systematically examines the mechanisms underlying the sensing and transduction of electrophysiological, mechanical, and biochemical signals in FGr-FET. We summarize strategies for enhancing FGr-FET sensing performance, including material modification, structural optimization, and the amplification of optical and magnetic signals. This paper highlights the latest advances in multimodal sensing and precision medicine applications and analyzes key challenges related to Debye screening, device reliability, inter-device variability, long-term operational stability, and clinical translation. Finally, we discuss the potential application of artificial intelligence algorithms in the precise extraction of physiological signals from FGr-FETs. These reviews provide a theoretical foundation for the development of next-generation precision medicine platforms.

## 1. Introduction

Technological advancements and interdisciplinary integration have driven significant developments in the field of biomedical detection. The field has evolved from traditional offline, intermittent detection to real-time, continuous, and in situ biosensing [1,2]. Field-effect transistors (FETs) are an excellent biosensing platform. They possess powerful signal amplification capabilities, ultra-low power consumption, and fast response times. These characteristics effectively overcome key limitations such as weak signal interference and low detection efficiency. FETs can amplify weak bioelectrical signals by 10^2^ to 10^5^ times, enabling highly sensitive detection of biomarkers at femtomolar to attomolar concentrations [3,4]. FETs consume only a few microwatts of power, making them ideal for wearable and implantable biosensing devices. Their fast response eliminates cumbersome experimental procedures and lengthy detection times, enabling real-time biosensing within seconds [4,5].

Graphene has become an ideal material for high-performance flexible FET devices due to its excellent mechanical flexibility, ultra-high carrier mobility and good biocompatibility. It has a Young’s modulus of up to 1100 GPa and a fracture strength of 42 N/m. Its excellent elasticity allows it to withstand repeated bending and folding deformation in flexible electronic systems [6]. The transport mobility of graphene at room temperature exceeds 1.5 × 10^5^ cm^2^/(V·s), which can be further increased to 10^8^ cm^2^/(V·s) through adjacent gate engineering and low temperature, matching the record transport mobility of traditional semiconductor heterojunctions [7]. A large number of studies have confirmed the good biosafety of original graphene [8]. It can effectively promote cell adhesion and proliferation without damaging mitochondrial membrane potential, cell morphology or autophagy activity, and it does not induce cell stress. These biological advantages are conducive to the construction of stable long-term neural and non-neural biological interfaces, making graphene very suitable for implantable flexible field-effect transistor (FET) biosensors [9]. Graphene-derived materials, including graphene oxide (GO) and reduced graphene oxide (rGO), also have attracted much attention due to their scalable fabrication processes and abundant surface functional groups [10,11]. In summary, these superior physical and biological properties make graphene a core functional material for high-performance flexible FETs, greatly enhancing its application potential in the biomedical field.

The integration of flexible electronic devices, FETs, and graphene has made it possible to fabricate FGr-FETs [12]. FGr-FETs combine the excellent physicochemical properties of graphene with the structural advantages of FETs. combine the excellent physicochemical properties of graphene with the structural advantages of FETs. Through field effects, piezoresistive effects, and charge modulation, they can perform multidimensional detection of biochemical, electrophysiological, and biomechanical signals, exhibiting excellent multimodal sensing performance. This paper systematically elucidates the sensing and conversion mechanisms of FGr-FETs for various biological signals and summarizes performance optimization strategies, including material modification, structural engineering, and signal enhancement assisted by optical or magnetic fields. We also review the latest progress of FGr-FETs in multimodal sensing and precision medicine and analyze key challenges related to Debye screening, device reliability, inter-device variability, long-term operational stability, and clinical translation. Finally, we explore the potential of artificial intelligence algorithms in accurately extracting physiological signals from FGr-FET outputs, providing theoretical support for the development of next-generation precision medicine platforms.

## 2. GFET Sensing Mechanism

**Figure 1:**
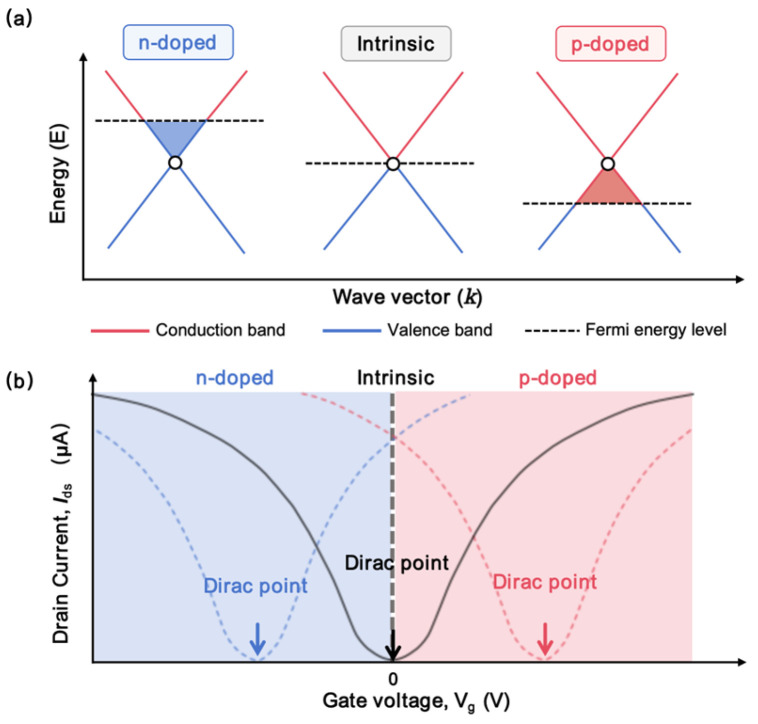
Schematic showing the GFET sensing mechanism. (**a**) Schematic representation of graphene Fermi energy level positions as a function of dopant. (**b**) Schematic representation of the changes in transfer curves/V_Dirac_ voltage of corresponding devices under different doping processes [14,15,16,17]. The arrow indicates the lowest point on the transfer characteristic curve.

Unlike conventional field-effect transistors (FETs), graphene field-effect transistors (GFETs) use a single-atom-thick layer of graphene as the channel. Linear performance dispersion occurs at the K and K’ points in the Brillouin zone, forming a Dirac cone, and the material has a zero-band gap (Figure 1a). In the intrinsic state shown in Figure 1a, the intersection of the conduction band (represented by the red line) and the valence band (represented by the blue line) is a point (circled in the figure), and the corresponding band gap is 0 (Fermi energy level represented by the dashed line in the figure). This electronic structure endows GFETs with intrinsic bipolar conductivity, exhibiting typical V-shaped or U-shaped transfer characteristic curves, which is the most significant difference between GFETs and conventional FETs [13,14]. The transfer characteristic curves are shown in Figure 1b, with intrinsic graphene corresponding to the black curve. The minimum drain voltage is the Dirac point, at which V_Dirac_ is 0 V.

The Fermi level can be continuously and reversibly tuned by adjusting the gate voltage, thereby switching between electron and hole transport. When Vg > V_Dirac_, the Fermi level rises to the conduction band, resulting in n-type conductivity; when Vg < V_Dirac_, the Fermi level falls to the valence band, resulting in p-type conductivity.

Environmental adsorption, substrate effects, and manufacturing impurities can cause spontaneous doping of graphene, leading to a Fermi level shift that causes V_Dirac_ to deviate from 0 V (Figure 1) [15]. Dopants with LUMO levels, the lowest unoccupied molecular orbital of the dopant molecule, the lowest empty energy level of the molecule, below the graphene Fermi level, remove electrons and induce p-type doping, shifting V_Dirac_ to a more positive potential (Figure 1b, light red area). Conversely, dopants with HOMO levels, the highest occupied molecular orbital of the dopant molecule, the highest empty energy level of the molecule, above the Fermi level, transfer charge to graphene and induce n-type doping, shifting V_Dirac_ to a more negative potential (Figure 1b, light blue area) [16]. The shift in the Dirac point is the operating principle of GFET sensors. Most devices work by quantifying biomolecules.

### 2.1. Fundamentals of Biochemical Sensing Based on the Field-Effect Characteristics of FETs

GFETs achieve reversible modulation of drain-source current by regulating the concentration and type of channel carriers through gate-induced electrostatic effects. External electrostatic disturbances on the graphene surface can serve as a virtual gate, with modulation capabilities comparable to traditional back-gate and liquid-gate modes [18]. Unlike bulk semiconductors, monolayer graphene confines all carriers within a single atomic layer, eliminating the bulk charge shielding effect. This gives GFETs atomic-level sensitivity to surface charge changes, with even minute molecular adsorption significantly altering channel carrier transport [14]. This superior surface charge sensitivity endows GFET-based biochemical sensors with ultra-high sensing performance.

Biomolecules immobilized on the graphene surface through specific adsorption or bioaffinity generate localized interfacial electric fields. These fields, acting as additional gate voltages, disrupt the original carrier balance. Negatively charged biomolecules induce hole accumulation in the graphene channels, while positively charged biomolecules induce electron accumulation. This redistribution of carriers shifts the Fermi level, resulting in a horizontal shift in the Dirac point voltage. Compared to current amplitude detection, the Dirac point shift reflects the overall carrier balance, thus providing greater stability and specificity against signal fluctuations [17,18].

**Figure 2:**
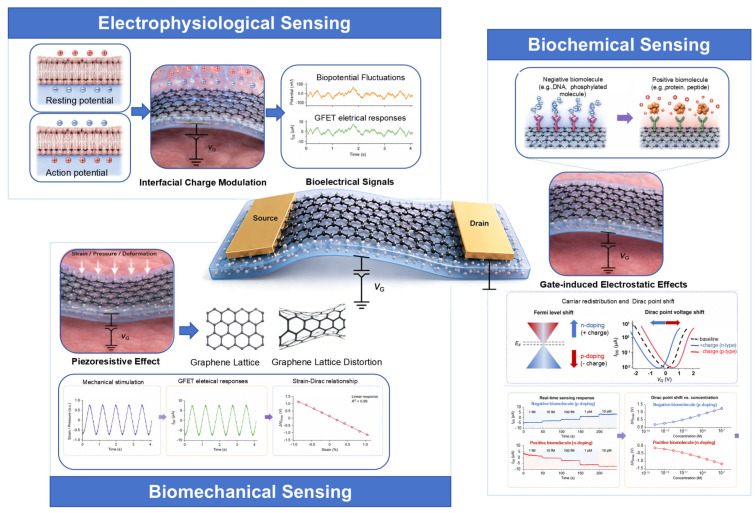
Schematic illustration of the FGr-FETs for multi-modal biosensing, including its working principle and representative results. Electrophysiological sensing is achieved through a localized electric field induced by biopotentials, which modulates carrier transport in the graphene channel. Biochemical sensing relies on biomolecule-induced redistribution of interfacial charge, leading to changes in carrier concentration and Dirac point voltage. Biomechanical sensing is achieved through strain-induced graphene lattice deformation, which alters carrier mobility and channel conductivity. These mechanisms work together to enable FGr-FETs for highly sensitive and multidimensional physiological monitoring.

In quantitative detection, the adsorbed biomolecules and induced interfacial charge are positively correlated with the analyte concentration [19]. Higher analyte concentrations lead to greater interfacial charge accumulation and a larger Dirac point shift. This linear relationship enables the establishment of quantitative calibration curves for precise biochemical detection, which has become the mainstream operating principle for GFET biosensors (Figure 2). Due to the inherent bipolar transport properties of graphene, this mechanism can be applied to biomolecules with positive and negative charges, including proteins, peptides, nucleic acids and small phosphorylated metabolites. It enables ultra-trace detection, even down to the zeptomole (zM) level [20].

### 2.2. Biomechanical Signal Sensing Mechanism Based on the Piezoresistive Effect Characteristics

Flexible GFETs based on the piezoresistive effect have significant application value in the field of biomechanical signal sensing, representing another important application in multidimensional biomedical sensing (Figure 2). The piezoresistive effect refers to the phenomenon where the resistance of a material changes significantly when subjected to mechanical stresses such as compression, tension, and bending. This change in resistance can be converted into an electrical signal and accurately detected [21,22].

When external mechanical stress (including skin stretching, vascular pulsation, and cell contraction) is applied, the flexible substrate deforms, driving synchronous deformation of the graphene channel [23]. This process causes graphene lattice distortion [24]. The lattice changes alter the transport paths and scattering probabilities of charge carriers within the channel, thereby increasing or decreasing carrier mobility and leading to significant changes in drain-source current. Mechanical deformation also affects the contact between graphene and the source/drain electrodes and the gate insulating layer [25]. Therefore, the electrical characteristics of the device are further modulated, resulting in more pronounced changes in the electrical signal. By monitoring the drain-source current in real time, the amplitude, frequency, and direction of the external mechanical stress can be inferred, enabling precise sensing of the mechanical signal.

Piezoelectric FGr-FET mechanical sensors offer significant advantages in biomedical applications. Their flexible structure allows them to conform closely to human skin and internal tissues without causing harm to the body. These devices consume low power, making them suitable for long-term real-time monitoring. With high sensitivity and fast response times, they can capture faint mechanical signals that are difficult for traditional sensors to detect.

To date, such sensors have been applied to human motion monitoring [26], measurement of physiological signals such as pulse and respiration [27], and cellular mechanics analysis [28], providing robust technical support for precision medicine. Current research is focused on optimizing device performance and expanding their applications to more complex biomedical scenarios.

### 2.3. Electrophysiological Signal Sensing Mechanism Based on Interface Charge Modulation Characteristics

Electrophysiological detection is one of the core biosensing applications of GFETs. Bioelectrical activity is generated by the directed migration of ions and transmembrane potential reversal within the cells [29]. This results in dynamic induced charges and local electric fields at the sensing interface. When a flexible GFET is tightly attached to a biological interface, these dynamic electric fields can act as in situ gate modulation. The inherent electron-hole balance in the graphene channel is continuously disrupted. Fluctuations in biopotential lead to the continuous accumulation and dissipation of interface charges. The Fermi level and carrier scattering behavior are dynamically modulated, causing synchronous changes in the Dirac point voltage and drain-source current (Figure 2).

Compared to traditional metal electrodes, graphene has no inherent surface states and possesses a large specific surface area [30]. Interface contact resistance is effectively reduced, and signal distortion and noise interference are significantly suppressed. GFETs can maintain high sensitivity to weak electrophysiological signals at the microvolt level [28]. Thanks to its flexible structure, the device can conform well to curved cell surfaces, soft tissues, and human skin. Non-invasive adaptive detection technology can be used to support long-term in situ biomonitoring.

This sensing technology is applicable to both microscopic and macroscopic electrophysiological signals. It can detect action potentials in single cells and neurons, as well as physiological signals such as electrocardiograms (ECG) and electromyograms (EMG) [31,32]. An integrated GFET array enables spatially resolved multi-channel synchronous signal acquisition. This technology provides reliable technical support for neurodynamic research, pathological analysis, and flexible wearable medical monitoring.

### 2.4. AI-Assisted Drawing of Figures

Some subplots in Figure 2 were generated using ChatGPT 5.5. Input prompts are as follows: Generate images of flexible graphite field-effect transistors in different signal sensing scenarios, demonstrating that electrophysiological sensing is achieved through a biopotential-induced local electric field that modulates carrier transport in graphene channels. Biochemical sensing relies on biomolecular-induced redistribution of interfacial charge, resulting in changes in carrier concentration and Dirac point voltage. Biomechanical sensing is achieved through strain-induced graphene lattice deformation, which alters carrier mobility and channel conductivity. The generated output is then processed using PowerPoint (Office 2021) for annotation, scaling, and formatting.

## 3. FGR-FET Sensor Performance Optimization Strategy

Conventional GFETs are fabricated on rigid silicon substrates. Replacing rigid substrates with a flexible substrate yields flexible graphene field-effect transistors (FGr-FETs), suitable for wearable and implantable biomedical applications. However, FGr-FETs face new performance challenges in flexible operating environments. Biological environments impose stringent requirements on device performance. Specifically, high sensitivity is required to detect weak biological signals, such as biomolecular interactions, electrophysiological activities, and subtle mechanical stimuli, which are often at low concentrations or microvolt-level amplitudes. Mechanical stability is essential to maintain reliable operation under repeated deformation, bending, stretching, or long-term use in dynamic biological environments. In addition, good biocompatibility is necessary to minimize cytotoxicity, inflammatory responses, and tissue damage while ensuring stable interfaces between the device and biological systems during prolonged monitoring. To maximize the potential of FGr-FETs in flexible biosensing, this paper summarizes a series of performance enhancement strategies, including graphene morphology manipulation, structural design optimization, interface engineering, and external field-assisted signal amplification. These methods effectively address the inherent limitations of traditional GFETs in terms of sensitivity, detection limit, and response rate. Therefore, the optimized FGr-FETs can meet the needs of complex and diverse biomedical sensing scenarios.

### 3.1. Graphene Morphology Optimization

Graphene morphology optimization is an effective strategy to improve the inherent electrical and mechanical properties of FGr-FETs. Planar graphene on rigid silicon substrates has high carrier mobility. However, its atomically flat surface has limited biomolecule binding sites and cannot withstand large mechanical deformations. Under flexible operating conditions, planar graphene is prone to cracks and defects under bending and stretching. These drawbacks can lead to a significant decrease in device performance and ultimately device failure. Currently, various methods have been developed to control the morphology of graphene. Wrinkled graphene structures have attracted much attention due to their ease of fabrication, significantly improved performance, and good compatibility with flexible device processes. The main fabrication methods include substrate pre-stretching [33], solvent induction [34] and template-assisted methods [33]. This type of structure can effectively improve the specific surface area and mechanical strain tolerance of graphene channels. The periodic wavy morphology of wrinkled graphene increases its channel-specific surface area compared to planar graphene [35]. The increased binding sites improve the charge trapping efficiency of biomolecules and the sensing sensitivity of the device. Furthermore, wrinkled graphene can modulate the formation of the electrical double layer and suppress the Debye shielding effect, further improving the sensing sensitivity and efficiency of the wrinkled FGr-FET [36]. Previous studies have demonstrated that wrinkled GFETs exhibit substantially enhanced sensing performance compared with planar devices. The sensitivity of wrinkled-interface GFETs was reported to be approximately one order of magnitude higher than that of flat counterparts, while three-dimensional wrinkled GFET biosensors achieved attomolar-level detection limits as low as 3.01 aM [35].

In terms of mechanical properties, the wrinkled structure buffers and disperses external mechanical stress through self-deformation. The maximum bending strain of the FGr-FET extends from 5% to over 20%. These structures greatly improve the flexibility and mechanical stability of the device, enabling it to adapt to complex wearable and implantable biomedical applications. Flexible GFETs also exhibit excellent mechanical durability, maintaining stable electrical performance after thousands to tens of thousands of bending cycles [37,38].

In addition to wrinkled structures, porous structures are another effective way to increase the specific surface area of graphene. Tunable-pore-size porous graphene can be synthesized via template methods, gas etching, and self-assembly [39,40,41]. This pore size range matches the size of common biomolecules such as proteins and nucleic acids. Although porous graphene has wide applications in electrochemical sensing, its use in field-effect transistor devices is limited.

This limitation is mainly attributed to the disruption of graphene’s inherent sp^2^ conjugated network. The formation of pores introduces numerous structural defects and edge states, thereby reducing carrier mobility. Furthermore, porous graphene can generate high leakage currents under liquid-gate conditions. This interference hinders the detection of target analytes, limiting the practical application of porous graphene in GFET biosensors.

### 3.2. Device Structural Design and Innovation

#### 3.2.1. Electrode Innovation of FGr-FETs

The modification of graphene can enhance the electrical sensing performance and mechanical flexibility of FGr-FETs. However, relying solely on the optimization of graphene will significantly limit the performance development of the device. To further improve the overall performance of flexible devices, flexible electrodes based on ultrathin metal films have been developed. By optimizing the thickness and deposition parameters of the metal film, mechanical flexibility is improved while maintaining high conductivity [42]. Currently, multilayer metal films, including Ti/Au and Cr/Au, are the most widely adopted electrode materials for FGr-FETs. Previous studies have demonstrated that properly designed metal thin-film electrodes can maintain stable electrical performance under repeated mechanical deformation, making them suitable for flexible and wearable GFET platforms [12,28].

The tensile properties of ultrathin metal films are limited, with a maximum tensile strain of less than 10%, which cannot meet the requirements of stretchable electronic devices. To overcome this mechanical limitation, novel flexible conductive materials have been developed, including liquid metals and silver nanowires. Eutectic gallium indium alloy (EGaIn) is a typical liquid metal with unique fluidity and a high electrical conductivity of about 3 × 10^6^ S/m, making it very suitable as an electrode for stretchable FGr-FETs. FGr-FETs based on EGaIn electrodes can still maintain stable electrical performance under tensile strains exceeding 50%, and the carrier mobility hardly decreases [43]. Silver nanowire networks have high conductivity, high transmittance, and excellent mechanical flexibility, achieving a low sheet resistance of 20 Ω/sq and a transmittance of over 90%. These networks can withstand tensile strains exceeding 20%. In addition, the integration of silver nanowires with graphene can effectively reduce contact resistance and improve the overall performance of the device [44]. These novel electrode materials effectively avoid the mechanical failure of traditional electrodes under flexible conditions. They have enriched the functional diversity of FGr-FETs and driven the development of stretchable, wearable, and implantable biosensors.

#### 3.2.2. Innovative Gate Structures of FGr-FETs

The gate is a core component that modulates carrier transport in an FGr-FET. It controls the device’s electrostatic control, sensing sensitivity, and anti-interference capability. The gate structure of FGr-FETs has evolved from traditional single-liquid-gate and back-gate designs to advanced configurations including dual-gate, extended-gate, and floating-gate structures.

The dual-gate design integrates independent back-gate and liquid-gate electrodes. The back gate precisely tunes the Fermi level of graphene to a high-sensitivity region near the Dirac point, while the liquid gate monitors potential changes caused by biomolecule binding. The dual-gate architecture amplifies the surface-potential signal through the capacitance ratio between the liquid gate and back gate, which can theoretically provide gains of 2–3 orders of magnitude [45]. To detect the miR-21 tumor marker, the sensitivity of GO/G-based SGGT using only a single solution gate was 19.26 mV/decade; with a dual gate, the sensitivity was significantly improved to 33.65 mV/decade due to gate-controlled doping achieved by the back gate [46].

The extended-gate structure physically isolates the transducer from the biorecognition region. Biofunctional modifications are performed only on the extended gate; thus, the graphene channel remains in its original state. This design effectively suppresses the non-specific adsorption of proteins and cell debris in complex biological fluids. The extended-gate architecture physically also separates the sensing interface from the transducer, thereby reducing signal drift. For example, Heshmat Asgharian et al. constructed a multi-electrode EGFET sensing platform by integrating a commercial MOSFET with a laser-induced graphene (LIG) extended gate, enabling the detection of vitamin C and SARS-CoV-2 antigen. In PBS solution, the platform achieved a detection sensitivity and detection limit of 73.67 mV dec^−1^ and 54.04 nM for vitamin C, respectively; in artificial saliva, the sensitivity and detection limit reached 81.05 mV dec^−1^ and 78.34 nM, respectively. Furthermore, the platform achieved a detection limit as low as 52 zg mL^−1^ for SARS-CoV-2 protein antigen. Long-term stability tests showed that, over a continuous 12-day test, the signal drift for vitamin C and SARS-CoV-2 detection was only 0.33 mV h^−1^ and 0.12 mV h^−1^, respectively, indicating that this extended gate structure possesses excellent long-term stability and anti-drift capability [47].

The floating-gate structure physically isolates the bio-recognition interface from the graphene channel, fundamentally eliminating the inherent trade-off between sensitivity, stability, and anti-interference performance commonly found in traditional liquid-gate Gr-FETs. Based on this structure, Wang et al. developed a split-type floating gate graphene transistor microfluidic biochip. This platform can simultaneously detect three liver cancer markers (CEA, AFP, and PTH) within 10 min, with a detection limit reaching the nanomolar level [48].

#### 3.2.3. Innovative Flexible Substrate Design of FGr-FETs

Substrate optimization plays a vital role in improving the performance of flexible GFET biomedical sensors. Suitable substrates are required to possess good biocompatibility, chemical stability and mechanical flexibility. The selection of substrate materials for FGr-FETs has been extensively investigated in previous studies [39]. In addition to material selection, reducing substrate thickness can also effectively improve the mechanical flexibility of the device. Polyimide (PI) and polyethylene terephthalate (PET) substrates with a thickness of less than 10 μm exhibit low bending stiffness. Flexible graphene field-effect transistors (GFETs) typically exhibit excellent mechanical stability due to the inherent flexibility of graphene and the application of flexible polymer substrates. Experimental studies have shown that flexible GFET devices can withstand bending radii as low as a few millimeters (equivalent to approximately 0.5% to 2% tensile strain) while maintaining stable electrical performance [49]. Furthermore, the research has confirmed their reliable operation under cyclic mechanical deformation; the devices retain their electrical properties after 1000 to 2000 bending cycles. For ultrathin GFET structures, even after repeated bending, they maintain over 90% of their initial mobility, highlighting their significant potential for long-term wearable and implantable biomedical applications [37].

Structural design is an effective method to improve the stretchability of flexible GFETs. Island-bridge structures consist of rigid GFET sensing units and stretchable metal interconnects. In this design, individual GFET devices are fabricated on rigid “islands,” while serpentine or meandering interconnects act as stretchable “bridges” to accommodate externally applied strain [50]. This mechanical decoupling strategy effectively isolates the active graphene channel from macroscopic deformation, thus maintaining the electrical properties of the GFET under mechanical bending and moderate tensile strain. This structure typically enables devices to operate stably at strains of a few percent and after thousands of bending cycles, making it ideal for wearable and implantable biosensing applications.

Serpentine source–drain electrodes adopt a planar spring configuration. This structure releases mechanical strain during large deformation and prevents electrode fracture. Yang et al. developed a similar advanced serpentine nested structure for graphene pressure sensors. It exhibits a resistance variation of merely 0.3% under 80% tensile strain, which effectively eliminates signal crosstalk between stretching and pressure detection [51]. Furthermore, Li et al. developed a mesh-like electrode structure, similar in shape to a spider web, which can be injected into a rabbit’s head for long-term in vivo monitoring of intracranial pressure and EEG signals [52].

### 3.3. Interfacial Modification and Optimization

Surface modification of graphene channels is a key method to improve the specificity and sensitivity of functionalized graphene field-effect transistor (FGr-FET) sensors. Sensing performance is enhanced by modulating the surface charge and interfacial electron transfer of graphene through the construction of specific recognition sites. The entire modification process involves three main steps: functionalization, bioreceptor immobilization, and site blocking. First, the clean graphene surface is modified with chemical groups or linker molecules to provide binding sites for bioreceptors. Then, bioreceptors with high targeting specificity, such as antibodies, aptamers, and enzymes, are immobilized on the graphene surface. Finally, residual active sites are blocked with bovine serum albumin (BSA) or polyethylene glycol (PEG). This treatment reduces non-specific adsorption and completes the surface modification of the device [53,54].

Graphene possesses inert and hydrophobic surfaces. Direct contact with biological samples triggers severe non-specific adsorption and signal interference. In addition, inefficient electron transfer between biomolecules and graphene channels impairs sensing sensitivity. Covalent functionalization is a widely adopted strategy for graphene modification. Stable covalent bonds are formed between graphene and organic molecules. This strategy offers excellent mechanical stability and is suitable for flexible device fabrication. Common covalent modification methods include amide coupling [55], diazonium-based grafting reactions, and electrochemical functionalization methods [56].

Click chemistry, proposed by K. Barry Sharpless in 2001, employs a modular principle to achieve efficient and stable molecular coupling and has become a mainstream technology for graphene functionalization [57]. Azide groups are anchored to graphene via diazonium salt electrochemical reduction or EDC/NHS activation. Alkyne-functionalized biomolecules react with a Cu(I) catalyst to generate stable triazole rings. This reaction can be completed within 1 to 2 h at room temperature. This method allows for precise control of surface modification density. The modified Gr-FET device maintains a carrier mobility above 1700 cm^2^/(V·s), which is beneficial for developing highly stable flexible biosensors [58].

Nevertheless, Cu(I) ions generated in the reaction exhibit biological toxicity. This issue restricts the application of copper-catalyzed azide-alkyne cycloaddition (CuAAC) in live-cell detection. In contrast, strain-promoted azide-alkyne cycloaddition (SPAAC) provides superior biocompatibility. It is applicable to live-cell imaging and in vivo biosensing. David Kaiser et al. developed a GFET biosensor based on a Van der Waals heterostructure consisting of graphene and an ultrathin azide-terminated carbon nanomembrane (CNM). DBCO-modified RNA aptamers were covalently immobilized on the CNM through SPAAC click chemistry, enabling highly specific detection of the inflammatory chemokine CXCL8/IL-8 in clinical samples. The ultrathin CNM served as a biofunctionalizable interposer while preserving the intrinsic electronic properties of graphene [59,60].

Non-covalent modification depends on physical forces, including π–π stacking, hydrophobic interactions and Van der Waals forces [61]. During this process, the graphene carbon network remains intact, thus preserving its inherent electronic properties well. However, this method is less stable than covalent modification. It is suitable for sensing applications requiring ultra-clean graphene surfaces. Pyrene and its derivatives are commonly used non-covalent modifiers. These aromatic molecules are adsorbed onto graphene through π-π stacking.

1-Pyrenebutyrate N-hydroxysuccinimide ester (PBASE) is a common bifunctional crosslinking agent. It binds to one end of graphene through π-π interactions. The terminal succinimide group can react with the amino group of biological receptors. Antibodies, aptamers and enzymes can be efficiently immobilized on graphene through this reaction. PBASE-modified GFET sensors can detect cardiac troponin I at concentrations as low as 10 pM, far below the clinical diagnostic threshold [62].

In addition, other graphene modification techniques have been reported for various sensing applications, including surface charge modulation [63], biomolecule functionalization, self-assembled monolayers, and polymer coatings.

Specific recognition sites are crucial for graphene surface engineering. Antibodies, aptamers, enzymes, and nucleic acid probes are modified onto GFETs to enable specific recognition of target biomolecules. This strategy supports the detection of biomarkers in complex samples such as serum, urine, and tissue fluid. A qualified recognition site must meet three core criteria: specific target binding, stable connection to graphene, and invariance to the electrical properties of graphene. Common modification methods include antibody conjugation, aptamer modification, and enzyme immobilization, each with unique biosensing advantages for different recognition molecules.

Antibodies are classic biosensing elements with high affinity and specificity for target antigens. Stable immune complexes can be formed effectively. Oriented antibody immobilization is adopted to fully expose antigen binding sites, thereby improving binding efficiency and sensing sensitivity. One search constructed a porphyrin-assisted GFET immunosensor with oriented antibodies. The sensor detects human epidermal growth factor receptor 2 (HER2) down to 1 pg/mL HER2 and maintains high selectivity in 10% human serum, demonstrating the potential of oriented biofunctionalization for high-performance GFET biosensing [64].

Aptamers are synthetic single-stranded nucleic acids or peptides. They feature small size, high stability and easy functional modification. Unique three-dimensional structures are formed for highly specific molecular binding. Wang et al. developed an aptamer-functionalized FGFET sensor. It exhibits excellent anti-interference capability in undiluted physiological samples and detects cervical cancer-related cytokines in cervical secretions, with a detection limit of 0.13 pM [65].

Enzyme modification utilizes specific enzyme-substrate catalytic reactions. Biological recognition behaviors are converted into readable electrochemical signals, which is suitable for the detection of small-molecule metabolites, including glucose and lactic acid. Nucleic acid probes identify target sequences based on base pairing, enabling sensitive detection of viral nucleic acids and gene mutations. Kong et al. fabricated a GFET functionalized with Y-shaped dual DNA probes. The sensor simultaneously targets the ORF1ab and N genes of SARS-CoV-2 and achieves a low detection limit of 0.03 copy μL^−1^. It accurately recognizes positive samples in five-in-one mixed detection with high specificity [66]. Precise functionalization of diverse recognition molecules enables FGFET biosensors to capture ultra-trace biomarkers in complex biological systems.

After the immobilization of biological recognition elements, unreacted sites on graphene surfaces are blocked with bovine serum albumin (BSA), polyethylene glycol (PEG) or zwitterionic polymers. This treatment effectively suppresses non-specific adsorption. To further optimize anti-interference performance, novel blocking methods have been developed. Guo et al. adopted a combined blocking strategy using hexamethyldisilazane (HMDS) and ethanolamine. Different from conventional polymer blocking, HMDS forms a dense hydrophobic monolayer to cover vacant surface defects. Ethanolamine quenches residual active functional groups on graphene. This dual chemical blocking eliminates non-specific binding thoroughly. This approach reduced non-specific signals by over 90% in 10% serum samples while retaining 1 fM ultrahigh sensitivity for Alzheimer’s disease biomarkers hsa-miR-125b and Aβ42. This blocking strategy significantly improves the overall sensing performance [67].

### 3.4. External Field-Assisted Signal Enhancement

Morphological and structural optimization has improved the basic sensing performance of FGr-FETs. Nevertheless, their detection limit and response speed are still constrained in complex biological fluids. Major challenges include the diffusion restriction of low-abundance biomolecules, Debye shielding and non-specific adsorption. External field-assisted strategies can regulate biomolecular transport and interfacial behaviors to overcome these inherent technical limitations.

Optical assistance enhances sensing performance via the photogating effect. When graphene is illuminated with light of a specific wavelength, electron-hole pairs are generated. Photogenerated carriers are trapped by interfacial defects and form a local electrostatic field. This field serves as an extra gate voltage and strengthens the conductance modulation of graphene channels.

Zhang et al. fabricated a photogate GFET sensor based on graphene/black phosphorus heterojunctions. Under 808 nm near-infrared irradiation, the sensor achieved a detection limit of 1 zM for the Alzheimer’s biomarker Aβ42, which was three orders of magnitude lower than that measured under dark conditions [21]. Furthermore, this light-induced signal amplification occurs within milliseconds, which effectively improves the overall sensing efficiency of Gr-FETs [68].

Magnetic-assisted strategies can effectively enrich target molecules and amplify sensing signals in complex biological samples by leveraging magnetic nanoparticle targeting and interfacial modulation. Superparamagnetic nanoparticles are modified with biological receptors. Driven by the magnetophoretic force of an external magnetic field, target molecules dispersed in the solution can be rapidly enriched on the graphene sensing region, achieving an enrichment efficiency of more than 95%. Liu et al. prepared two antibody-functionalized magnetic composites, Fe_3_O_4_@UiO-66-NH_2_ and Fe_3_O_4_@SiO_2_@Ce-Zr MOF, to capture SARS-CoV-2 antigens. When integrated with GFET detection, the system realized an ultra-low detection limit at the ag/mL level [69,70]. The spacing between magnetic nanoparticles and graphene surfaces can be precisely tuned by varying the magnetic field strength. When nanoparticles are positioned within the Debye length of graphene, their surface charges generate intense electrostatic modulation and induce significant shifts in the Dirac point. One study developed an GFET sensor based on this magnetic interfacial effect. The sensor achieved a detection limit of 10 pM for cardiac troponin I and retained excellent sensing stability in whole blood samples [62]. Compared with other external field-assisted methods, magnetic assistance has the unique advantages of non-contact operation, remote controllability and low biological background interference. It is highly applicable to implantable and wearable biosensing devices.

## 4. Applications of Flexible GFETs in Biomedical Sensors

Precision medicine requires rapid, accurate, highly sensitive, and highly specific detection of biomarkers. Traditional detection methods, such as enzyme-linked immunosorbent assay (ELISA) and polymerase chain reaction (PCR), suffer from drawbacks such as complex operation, long detection time, high detection limits, and reliance on bulky laboratory equipment. These limitations restrict their practical application in point-of-care testing (POCT) and early disease screening. FGr-FETs are widely adopted for detecting three primary types of diagnostic biomarkers: proteins, nucleic acids and metabolites. In the field of precision medicine, FGr-FET is also used for biomechanical and electrophysiological detection. Benefiting from the unique biochemical interface conversion capability and high-sensitivity charge response, FGr-FETs can effectively solve the above technical bottlenecks.

### 4.1. Protein Detection

Proteins are important biological macromolecules and key biomarkers for clinical diagnosis and disease monitoring. Low-abundance protein biomarkers, such as tumor markers and inflammatory factors, are easily affected by background interference in biological samples, thus affecting their accurate detection. Thanks to effective surface functionalization, FGr-FETs can specifically recognize target proteins through antibody conjugation and aptamer modification. Unlike traditional detection methods, FGFET sensors do not require complex sample pretreatment. This characteristic makes FGr-FETs suitable for early tumor screening and real-time monitoring of inflammatory states.

Antibody immobilization is a common and effective strategy for endowing GFETs with specific protein recognition capabilities. In related research, a flexible FET biosensor based on RGO for the detection of inflammatory biomarkers was constructed. The abundant and uniform carboxyl active sites on the surface of reduced graphene oxide allow for the stable and orderly covalent immobilization of the IL-6 antibody. This regular antibody arrangement ensures efficient antigen recognition and reduces interfacial nonspecific interference. 105In another representative work focusing on the detection of neurodegenerative diseases, Huang et al. modified a GO/G hybrid substrate with a high-affinity monoclonal antibody targeting Alzheimer’s disease-related proteins. Through uniform surface chemical activation, the antibody was firmly anchored to the graphene interface, thus maintaining stable biorecognition activity. The fabricated GFET sensor achieved quantitative detection of plasma tau protein and phosphorylated p-tau217 protein by recording the Dirac point shift in the graphene channel, with a linear range of 10 fg/mL to 100 pg/mL. Furthermore, the sensor maintained excellent specificity and stability even in the complex human serum environment [71].

Aptamer-based recognition is an alternative and widely studied strategy for protein detection using graphene GFETs. Aptamers are artificially selected single-stranded DNA or RNA oligonucleotides that have high affinity and specificity for target proteins. Compared with antibodies, aptamers have advantages such as better stability, lower production cost, and easier surface modification, making them ideal recognition elements for constructing flexible sensing interfaces [72]. In a representative study, researchers constructed a flexible aptamer sensor based on reduced graphene oxide (rGO) GFETs for detecting brain-derived neurotrophic factor (BDNF). The aptamer was stably immobilized on the rGO surface by PBASE crosslinking. The sensor achieved a low detection limit of 0.4 nM. Its sensing performance remained stable after repeated bending, verifying the excellent mechanical reliability of aptamer-based flexible biosensors in wearable and long-term monitoring [71]. Thanks to the stable immobilization of aptamers, oligonucleotide sequences can specifically bind to target proteins, achieving accurate biorecognition. Combined with HMDS hydrophobic blocking, the non-specific adsorption of the sensor can be significantly reduced, further improving the anti-interference ability. This integrated strategy enables fM-level ultrasensitive detection of the Alzheimer’s disease biomarker Aβ42, demonstrating significant advantages in the detection of low-abundance neuroproteins [67].

Furthermore, molecular imprinting technology provides a probe-free identification method. Specific protein imprint membranes are constructed on graphene surfaces for targeted capture of biomarkers. This method overcomes the limitations of traditional biological probes and improves detection sensitivity to the aM level, providing a new avenue for developing long-term stable biosensing devices [73].

### 4.2. Nucleic Acid Detection

Nucleic acids carry the genetic information of all organisms, and their detection can be used for disease risk assessment and targeted prevention [74]. GFETs can bind to nucleic acid probes and identify target sequences through specific molecular hybridization. The charge change during hybridization modulates the carrier density of the graphene channel, thus enabling highly sensitive nucleic acid detection without amplification pretreatment [75].

Traditional graphene field-effect transistors suffer from severe signal interference in aqueous environments. Water molecules disrupt the Fermi level of graphene, and the Debye shielding effect further weakens the sensing signal. To overcome these drawbacks, Yang et al. prepared a molybdenum disulfide/graphene (MoS_2_/graphene) hybrid field-effect transistor sensor for ultrasensitive DNA detection [76]. The MoS_2_ layer acts as a protective barrier, eliminating aqueous noise. At the same time, its unique charge distribution polarizes DNA molecules, shortens the distance between DNA and the sensing interface, and enhances the DNA donor effect. Compared with the weakened gate effect, the dominant donor effect promotes electron transfer and causes a significant Dirac point shift. This hybrid structure effectively mitigates the interference of the Debye shielding effect, achieving an ultra-low detection limit of 10 aM.

Furthermore, the combination of GFET with gene editing technology greatly simplifies nucleic acid detection. Kiana integrated GFET devices with a CRISPR-Cas9 system. This method is used to identify unamplified target genes and single-point DNA mutations in complex biological samples [77,78]. This technology avoids cumbersome extraction, purification, and amplification steps, achieving high sensitivity of one copy per 100 μL, suitable for rapid clinical disease screening [79].

It is noteworthy that the ultra-high sensitivity of reported CRISPR-integrated GFET biosensors is not entirely attributable to the GFET’s conversion mechanism. The contribution of the CRISPR system to sensing performance varies significantly across different CRISPR-GFET architectures. In CRISPR chips employing immobilized dCas9 [77], signal generation is primarily attributed to target-induced charge modulation and GFET electro-signal conversion, without enzymatic amplification. In contrast, Cas12 [80] and Cas13-based [81,82]. GFET platforms benefit from their inherent cleavage activity, providing an intrinsic biochemical amplification mechanism and significantly improving previously reported ultra-low detection limits. Therefore, the overall sensitivity reflects the synergistic effect of the CRISPR reaction and GFET electro-signal readout. Although graphene field-effect transistors (GFETs) enable label-free, real-time, and highly sensitive electro-signal conversion, the enzymatic amplification introduced by the CRISPR system may further reduce the detection limit.

### 4.3. Metabolite Detection

Enzymatic sensing is a key strategy for rapid metabolite detection using FGr-FETs. Metabolic oxidases are immobilized on graphene surfaces. Specific catalytic reactions between enzymes and target metabolites alter interfacial charge distribution and modulate FGr-FET electrical signals. For example, Zhi et al. immobilized glucose oxidase on an ultrathin affinity-gated graphene transistor to construct a flexible glucose biosensor. Benefiting from the enzymatic field-effect sensing mechanism and the ultrathin device architecture, the sensor achieved a detection limit of 0.1 μM, exhibited excellent selectivity toward glucose, and maintained stable responses during repeated measurements. Furthermore, successful detection in tear and sweat samples demonstrated its suitability for wearable and noninvasive glucose monitoring applications [83]. Enzymatic methods can also detect lactate [84] and cholesterol [85] but have not yet been integrated with FGr-FET. In contrast to enzymatic sensing, non-enzymatic recognition techniques are adopted for the detection of other metabolic biomarkers. Antigen–antibody binding and aptamer-based sensing enable fast detection of cortisol from tens of seconds to several minutes [86], while ferritin detection requires 5–15 min due to specific immune binding reactions [87]. These FGr-FET sensors have excellent flexibility and biocompatibility and can be combined with flexible films and contact lenses to enable rapid monitoring of metabolites in human tears [86], providing promising technical support for the development of advanced wearable health monitoring devices.

Table 1 summarizes typical applications of flexible graphene field-effect transistor (FGr-FET) biosensors in the detection of proteins, nucleic acids, and metabolites reported over the past five years. This table compares the graphene materials, flexible substrates, surface functionalization strategies, sensing targets, detection mechanisms, and analytical performance of different FGr-FET platforms. Various biorecognition elements, including antibodies, aptamers, enzymes, and nucleic acid probes, have been used to achieve selective and sensitive detection of clinically relevant biomarkers.

Although many studies have reported extremely low limits of detection, directly comparing sensing performance remains challenging due to significant differences in experimental conditions, sample matrices, device structures, and definitions of the limit of detection across different studies. Therefore, in addition to analytical sensitivity, Table 1 also summarizes the specificity, operational stability, and real-world sample validation of the sensors to provide a more comprehensive assessment of device performance. The results show that many FGr-FET biosensors exhibit excellent selectivity for target analytes, maintain stable sensing performance under repeated measurements or mechanical deformation, and have been successfully validated in clinically relevant samples, including serum, saliva, sweat, tears, urine, and other biological fluids.

These findings demonstrate the immense potential of FGr-FET biosensors for wearable health monitoring, point-of-care diagnostics, and precision medicine. However, challenges remain to be addressed before widespread practical application, including performance standardization, long-term stability, large-scale reproducibility, and clinical validation.

### 4.4. Biomechanical Signal Monitoring

FGr-FETs have shown significant advantages in the field of physical sensing. The synergistic effect of piezoresistive response and field-effect modulation makes them widely used in biomechanical detection [97]. Flexible strain sensors based on FGr-FETs can be closely attached to human skin for physiological monitoring. When fixed to the radial artery, they can detect changes in pulse rate at rest and after exercise; when attached to the outer canthus of the eye, they can reliably record blinking [98]. Paul developed a biodegradable FGr-FET pressure sensor using a 3 μm thick flexible substrate and a wrinkled graphene network structure. This structure can achieve a pressure detection range of 0.5–2 kPa without additional modification. The sensor can continuously capture subtle movements of the temporalis muscle and enable non-contact control of robotic devices [99].

With its ultra-high sensitivity, FGr-FETs are suitable for advanced basic medical and implantable sensing applications. These devices can detect changes in the contractility of individual cells, which is crucial for studying the physiological function of cardiomyocytes and promoting drug screening. One study prepared a graphene integrated mesh electronic system. This system can simultaneously record the electrical signals and mechanical contraction dynamics of cardiac microtissues, realizing multimodal monitoring of excitation-contraction coupling. It provides a reliable tool for exploring the pathological mechanisms of heart disease and developing novel therapeutic drugs [100]. Due to its ultrathin structure, good flexibility and excellent biocompatibility, FGr-FET can be implanted in the body to monitor the dynamic mechanical changes in organs [52].

### 4.5. Electrophysiological Signal Sensing

Electrophysiological signals reflect the activity of the human nervous and muscular systems. These signals range from microvolt-level neuronal potentials to millivolt-level cardiac signals, carrying crucial physiological and pathological information. FGr-FETs possess excellent electrical and mechanical properties, making them a powerful tool for high-precision electrophysiological monitoring. In implantable neural interface research, Andrea developed a flexible FGr-FET system based on polyimide (PI) films and bilayer metal electrodes. This device can achieve close adhesion to the mouse cerebral cortex. It is equipped with 14 recording sites, which can maintain stable signals during head movements and record abnormal epileptiform discharges in mice, providing a new strategy for the diagnosis and treatment of epilepsy [101].

FGr-FETs have an ultrathin structure, excellent flexibility, and good biocompatibility. They can form a stable long-term interface with neural tissue and reduce inflammation and tissue damage caused by traditional rigid electrodes. One study designed a mesh-like 16-channel FGr-FET neural array. After cortical implantation, the device can continuously record the action potentials of a single neuron for up to 5.5 months with minimal signal attenuation. The induced glial cell proliferation is only one-fifth that of traditional silicon electrodes [102]. This highly stable and high-resolution recording technology has facilitated the development of brain–computer interfaces and enabled precise external device control for paralyzed patients. In terms of cellular electrophysiological detection, FGr-FET can achieve high spatial resolution recording of the electrical activity of cardiomyocytes and neurons. Benno et al. prepared a high-density FGr-FET array that can monitor the action potential changes in HL-1 cardiomyocytes at 36 sites in real time. This platform supports cardiac pathology research and new drug screening [28].

## 5. Challenges, Limitations, and Future Trends in FGr-FETs

The preceding article systematically reviewed the working mechanisms of FGr-FETs in charge modulation, interface coupling, and multimodal signal conversion from the perspective of multidimensional physiological signal sensing. This article comprehensively summarizes performance enhancement strategies such as material modification, structural design, and signal amplification. The following section will further explore current research hotspots and key challenges, including device reliability, the Debye shielding effect, clinical translation, and AI-assisted data analysis.

### 5.1. Debye Shielding Effect and Device Design Trade-Offs

FGr-FET biosensors possess extremely high sensitivity, but the Debye shielding effect remains a fundamental physical bottleneck limiting their application under physiological conditions.

Most biorecognition molecules, including antibodies and protein receptors, are much larger. The charge change induced by target binding decays significantly before reaching the graphene channel, thus limiting the effective sensing window of the device in pristine biological samples. Two main approaches have been proposed to mitigate this shielding limitation.

The first approach aims to reduce the Debye shielding effect by shortening the distance between the charged active biological layer and the graphene channel, thereby improving signal coupling efficiency. Researchers can achieve this by employing compact recognition motifs (aptamers, nanobodies [103,104], short peptide ligands) and thin biofunctional coatings, as well as interface engineering strategies such as π-π stacking, short-chain self-assembled monolayers, and direct surface immobilization. These methods can improve signal transduction in complex biological fluids, including serum, saliva, and interstitial fluid.

A second approach is to optimize channel structure and interfacial electrical environment to improve device electroresponsiveness and signal readout performance. For example, two-dimensional heterostructures (MoS_2_, h-BN) can be integrated into -Gr-FETs to enhance gate coupling and signal amplification. Furthermore, ultrathin dielectric layers or ion-barrier layers can be introduced to stabilize the device and suppress non-specific charge interference.

However, these structural improvements introduce inherent performance trade-offs. Dielectric layers tend to reduce gate coupling efficiency and transconductance. Heterostructures increase interfacial resistance and suppress carrier mobility, while also increasing fabrication complexity and reducing device uniformity.

In short, strategies to mitigate the Debye shielding effect should not merely pursue high sensitivity but should be viewed as a multi-parameter synergistic optimization challenge. Next-generation flexible graphene field-effect transistors (FGr-FETs) must achieve a holistic balance between sensitivity, stability, electrical performance, mechanical compliance, and manufacturability. A quantifiable performance benchmark system should be established to simplify the translation process from laboratory prototypes to deployable biomedical sensing platforms.

### 5.2. Equipment Reliability, Variability, and Long-Term Stability in Practical Application Scenarios

The excellent detection limits of reported FGr-FET biosensors often mask another equally important issue: the reproducibility of sensing performance under practical application conditions. Femtomolar or even attomolar detection performance achieved in laboratory conditions is typically influenced by a variety of factors, including the source of graphene materials, device fabrication processes, interface functionalization strategies, and testing environments and data processing methods. In flexible wearable applications targeting real physiological environments (e.g., sweat, tissue fluid, or continuous glucose monitoring systems), the application scenarios are even more complex and varied. Besides sensitivity, inter-device variability, batch-to-batch consistency, and long-term stability remain key factors limiting the practical application of FGr-FET biosensors.

Most reported device performance is still based on small-batch, laboratory-scale fabrication, and batch-to-batch variability or different fabrication processes can lead to significant differences in device performance. For example, chemical vapor deposition (CVD) graphene typically possesses high carrier mobility and low intrinsic defect density, but its transfer process is prone to introducing cracks, polymer residues, and interface contamination. While reduced graphene oxide (rGO) offers good solution processing compatibility and low cost, the distribution of residual oxygen-containing functional groups and defects within its structure is difficult to control precisely. Laser-induced graphene (LIG) possesses excellent flexible processing capabilities and scalable manufacturing potential, but its porous network structure and degree of local graphitization typically exhibit significant spatial inhomogeneities. These material-level differences further propagate to the device level, manifesting as significant fluctuations in Dirac point location, transconductance, noise level, and sensing response amplitude.

Furthermore, even using the same graphene material system, factors such as photolithography precision, electrode contact quality, surface functionalization density, and bio-feature element immobilization efficiency during device fabrication introduce additional uncertainties, leading to significant variations in device performance. In the fabrication of flexible devices, substrate surface roughness, stress release behavior, and the consistency of material transfer processes are difficult to strictly control. These inter-device and batch-to-batch variations are typically more significant than those of traditional rigid silicon-based field-effect transistors (FETs). Therefore, the ultra-low detection limits and high sensitivity results reported in the literature may not reflect the true performance of devices under large-scale production conditions. Establishing unified manufacturing standards, statistical evaluation methods, and batch device performance reporting specifications remains a key issue for the practical application of flexible graphene field-effect transistors (FGr-FETs).

Besides repeatability issues, long-term stability is also a critical factor limiting the practical application of FGr-FETs, especially in scenarios requiring long-term operation, such as continuous health monitoring and implantable biosensors. Device signal drift typically stems from multiple coupling effects, including charge trapping and release at the graphene/flexible substrate interface, gradual deactivation or remodeling of the biofunctional layer, non-specific adsorption in complex biological environments, and cumulative mechanical strain caused by repeated bending, stretching, or attachment. These factors collectively affect the charge transport behavior of the graphene channel, leading to Dirac point drift, transconductance variations, and baseline signal instability. This significantly reduces the quantitative accuracy of the device in continuous monitoring scenarios.

While strategies such as hydrophobic/antifouling coatings, packaging structure design, and flexible interface stress modulation have improved device stability to some extent, achieving stable operation under dynamic mechanical deformation and long-term coupling with complex physiological media remains challenging. Therefore, improving the performance of flexible FGr-FET biosensors should not only focus on their limiting sensitivity but also incorporate multi-dimensional parameters such as noise level, signal drift rate, cycle stability, device consistency, and long-term continuous monitoring capability in real biological fluids into the device design and manufacturing considerations. This will drive FGr-FETs from the laboratory proof-of-concept stage to practical biomedical applications.

### 5.3. Challenges in Clinical Translation and Practical Applications

Besides the inherent limitations of the devices themselves, the complexity of real-world clinical applications also poses challenges to the practical application of FGr-FETs. Most current research is still based on limited sample sizes and controlled experimental conditions, and performance evaluation typically relies on validation using standard buffers, artificially simulated samples, or small-scale clinical samples. However, the concentration distribution of biomarkers in real-world clinical settings is often influenced by multiple factors such as age, sex, genetic background, disease stage, drug use, and metabolic state, exhibiting significant individual differences and population heterogeneity. Therefore, even if an FGr-FET platform demonstrates excellent sensitivity and specificity under laboratory conditions, its detection performance still needs to be validated through multi-center, large-sample, and long-term follow-up cohort studies to fully demonstrate its clinical applicability and universality [105].

Meanwhile, clinical translation depends not only on the detection performance itself but also on its ability to create additional value within the existing healthcare system. For the vast majority of disease diagnostic scenarios, methods such as enzyme-linked immunosorbent assay (ELISA), polymerase chain reaction (PCR), digital PCR, and liquid chromatography-mass spectrometry (LC-MS/MS) remain the currently recognized clinical gold standard. This means that novel FGr-FET sensors not only need to demonstrate good correlation and consistency with existing standard methods but also need to answer a more critical question: whether they can provide clinical information that is difficult to obtain using traditional methods. For example, continuous dynamic monitoring capabilities, real-time disease progression tracking, treatment response assessment, and long-term outpatient health management are the core advantages that truly distinguish wearable and implantable sensing platforms from traditional in vitro detection technologies. In fact, in recent years, the development trend in the field of wearable biosensors has gradually shifted from simply pursuing detection limits and response speeds to building digital biomarkers with clinical decision-making value, emphasizing the acquisition and interpretation of continuous, multidimensional, and personalized health data [106].

Furthermore, the current evaluation system for FGr-FET biosensors still lacks a unified standard. Many studies use different sample sources, preprocessing methods, detection conditions, and performance indicators, making it difficult to conduct cross-platform comparisons. While detection limit, linear range, and response time are important parameters, these metrics do not fully reflect the actual value of devices in real-world clinical settings. For continuous monitoring systems, long-term stability, drift rate, batch consistency, patient compliance, false alarm rate, and clinical endpoint prediction ability are often equally or even more important. Future development requires establishing a standardized evaluation framework similar to that used in the continuous glucose monitoring (CGM) system field, incorporating analytical performance, biocompatibility, clinical effectiveness, and patient benefit into a unified evaluation system. This will promote the formation of comparable, reproducible, and regulated evaluation standards across different studies.

Besides technical and clinical validation issues, regulatory requirements also constitute a significant hurdle for practical translation. Unlike laboratory prototypes, FGr-FET systems for clinical applications need to meet the requirements of medical device regulatory agencies regarding safety, effectiveness, biocompatibility, data integrity, and cybersecurity. For implantable or long-term attached devices, systematic toxicological evaluations, long-term stability testing, and real-world evidence studies are also necessary. As wearable devices gradually become an important part of the digital healthcare ecosystem, the large-scale continuous health data generated by sensors will also raise new issues such as data privacy protection, algorithm transparency, and the determination of medical liability. Therefore, the clinical translation of FGr-FET biosensors is not only a matter of materials science and device engineering but also an interdisciplinary systems engineering project involving clinical medicine, regulatory science, digital health, and health economics. Only by achieving breakthroughs in key areas such as large-scale clinical validation, the establishment of standardized evaluation systems, and the clarification of regulatory pathways can flexible graphene biosensing technology truly achieve the leap from laboratory prototype to clinical practice.

### 5.4. AI-Driven Future Trends in FGr-FET Biosensors

FGr-FETs possess atomic-level thickness, ultra-high carrier mobility, and excellent mechanical flexibility. Leveraging the in situ signal amplification capability of field-effect transistors, FGr-FETs effectively overcome the limitations of traditional biosensors in terms of detection sensitivity, response speed, and biointerface compatibility. These sensors can perform high-precision, multi-dimensional detection of biochemical, biomechanical, and electrophysiological signals. By optimizing material morphology, device structure, interface engineering, and external field modulation, the detection limit of FGr-FETs has reached the zeptomole level. Their mechanical stability and anti-interference capabilities have also been significantly improved. Therefore, FGr-FETs show broad application prospects in precision medicine, covering areas such as tumor marker detection, viral nucleic acid screening, wearable physiological monitoring, and implantable neural sensing.

Artificial intelligence is gradually becoming a powerful tool for optimizing the sensing performance of FGr-FET systems. Random forest-based machine learning models can effectively calibrate manufacturing deviations and long-term signal drift between FGr-FET devices. In calcium ion detection experiments, this model achieved a prediction accuracy of up to 0.999, outperforming traditional algorithms including linear regression, support vector machines, and decision trees [107]. This method provides a reliable technical guarantee for the high accuracy and stable operation of FGr-FET sensors.

Currently, FGr-FET biosensors are developing towards multimodal integration, enabling a single device to simultaneously acquire biochemical, biomechanical, and electrophysiological signals. However, the electrical properties of graphene are susceptible to external interferences such as temperature, humidity, and mechanical strain, leading to severe signal crosstalk and decreased detection accuracy. Artificial intelligence provides an effective solution to this bottleneck problem. Machine learning and deep learning algorithms can extract hidden feature information from massive amounts of raw sensor data and establish nonlinear correlations between raw signals and target parameters [108]. These technologies enable precise separation of interference signals, synchronous decoupling of multiple detection parameters, and intelligent decoding of complex physiological signals [109].

The deep integration of artificial intelligence and FGr-FET represents the core development trend of intelligent biomedical sensing. This integrated system supports real-time acquisition, precise processing, and rapid analysis of sensor data, greatly improving the intelligence level of FGr-FET sensors [103]. By combining big data technology, systematic analysis and mining of sensor data from multiple scenarios can be performed. This technical framework provides comprehensive and reliable data support for precision medical diagnosis and personalized health management.

## Figures and Tables

**Table 1:** Summary of biochemical sensing applications of FGrFETs in recent five years.

Type	Year	Graphene Material	Flexible Substrate	Modification Method	Sensing Substance	Detection Method	Detection Target	Detection Sensitivity	Specificity	Stability	Real Sample Validation	Reference
Protein	2026	Drop-casting Reduced Graphene Oxide (rGO)	Glass/PET	GO-rich hairpin probes + Anti-IL-6 antibody	Anti-IL-6 antibody	Antibody-coupled recognition	IL-6	0.022 pg/mL	Good anti-interference performance toward coexisting cytokines	Maintained performance over 21 days and across 3 batches	Human serum from sepsis patients	[88]
	2025	Reduced Graphene Oxide (rGO)	Polyimide (PI) film	PBASE-assisted aptamer immobilization	BDNF aptamer	DNA aptamer recognition	BDNF	0.4 nM (linear range 0.025–1000 nM)	~1% response to 10 μM interferents	Signal decreased ~7% after 5 days and ~21% after 30 days	Complex biological samples	[71]
	2021	CVD Graphene	Mylar (2.5 μm)	Pyrene linker + aptamer	Nucleic acid aptamer	Aptamer-GFET	TNF-α	5:00 PM	High selectivity against non-target proteins	Flexible operation maintained under deformation	Biofluid validation reported	[89]
	2022	CVD Monolayer Graphene	PET	Pyrene linker + aptamer	Nucleic acid aptamer	Aptamer-coupled recognition	Hemoglobin (Hb)	10.6 fM	Excellent discrimination against interfering proteins	Stable response after repeated measurements	Human blood sample validation	[90]
	2022	Monolayer Graphene	Polyimide	PBASE + aptamer immobilization	Nucleic acid aptamer	Aptamer-GFET	IL-6	10 pM–100 nM	High specificity toward IL-6	Stable operation under bending conditions	/	[91]
	2025	Monolayer CVD Graphene	PET flexible film (1 μm)	Antibody immobilization (Au–S linkage + crosslinking)	Ferritin antibody	Antibody-coupled recognition	Ferritin	LOD = 27 ng/L	Negligible response to non-target proteins	Stable sensing performance during repeated tests	Serum sample validation	[87]
	2024	Reduced Graphene Oxide (rGO)	PET	rGO coating + antibody immobilization	PSA-specific antibody	Antibody-coupled recognition	PSA	Real-time detection, fM-level sensitivity	High selectivity toward PSA: in the presence of a very high BSA concentration (0.5 × 10^−6^ M), the LOD of target PSA remained 1.7 × 10^−15^ M	Long-term stability reported	Bovine serum albumin (BSA) mixed samples	[92]
Nucleic Acid	2026	Drop-casting rGO	Glass/PET	DNA probe-functionalized GO/rGO-FET	DNA probe	Complementary base pairing	ssDNA	3.98 fM	Not reported	Withstood three 95 °C thermal cycles and four regeneration cycles in 8 M urea	Not reported	[88]
	2021	Liquid-phase exfoliated graphene (LEG)	PET film	DNA methylation-sensitive probe immobilization	DNA	Hybridization-induced GFET response	DNA methylation biomarkers	LOD not reported	Able to distinguish methylated and non-methylated DNA sequences	Disposable sensor showed stable single-use performance; long-term stability not reported	Not reported	[93]
	2024	Monolayer CVD graphene	PDMS flexible elastomer (LIG electrode)	PASE crosslinking + PLL immobilized probe DNA	DNA probe and ssDNA	Label-free hybridization + CRISPR/Cas12a amplification	ssDNA, viral RNA	10 fM (without amplification); 1 fM (CRISPR enhanced)	Excellent discrimination against non-target sequences and single-base mismatches	Stable operation after repeated measurements; bending stability demonstrated	Viral RNA samples and spiked biological samples	[94]
	2025	Monolayer CVD graphene	PDMS flexible elastomer (LIG electrode)	AuNP modification + thiol hairpin DNA probe	DNA probe and ssDNA	CRISPR-Cas10 coupled GFET (amplification-free)	SARS-CoV-2 RNA, miRNA	0.1 fM; single-copy detectable/100 μL	High specificity enabled by CRISPR-Cas10 target recognition; negligible response to non-target RNA	Not reported	Clinical RNA samples and complex biological matrices	[79]
Metabolite	2020	Monolayer CVD Graphene	PI ultrathin film	PDA + GOx	Glucose Oxidase	Enzymatic field effect	Tear/sweat glucose	LOD 0.1 μM	High specificity toward glucose	Stable during repeated measurements	Tear and sweat samples	[95]
	2022	rGO	PET	Electropolymerized PABA + GOx	Glucose Oxidase	Enzymatic field effect	Urine glucose	LOD 4.1 μM	Good selectivity against urinary interferents	Stable over repeated testing	Human urine samples	[96]

## Data Availability

No new data were created or analyzed in this study. Data sharing is not applicable to this article.

## Abbreviations

The following abbreviations are used in this manuscript:

FET	Field-Effect Transistor	rGO	Reduced Graphene Oxide
FGr-FET	Flexible Graphene Field-Effect Transistor	LIG	Laser-Induced Graphene
463	Graphene Field-Effect Transistor	SAM	Self-Assembled Monolayer
AP	Action Potential	PBASE	1-Pyrenebutyrate N-Hydroxysuccinimide Ester
PI	Polyimide	BSA	Bovine Serum Albumin
PET	Polyethylene Terephthalate	PEG	Polyethylene Glycol
PDMS	Polydimethylsiloxane	HMDS	Hexamethyldisilazane
ITO	Indium Tin Oxide	SPAAC	Strain-Promoted Azide-Alkyne Cycloaddition
EGaIn	Eutectic Gallium-Indium Alloy	2D	Two-Dimensional
SSDNA	Single-stranded DNA	CRISPR	Clustered Regularly Interspaced Short Palindromic Repeats
Cas	CRISPR-Associated Protein	LOD	Limit of Detection
fM	Femtomolar	SNR	Signal-to-Noise Ratio
aM	Attomolar	AI	Artificial Intelligence
zM	Zeptomolar		

## References

[1] Ling S., Xu C., Jiang Y., Xu F., Jin H., Chen H., Chen S., Ding Z., Sun D. (2026). Toward Continuous Monitoring Systems: Emerging Trends of on-Chip Sensors in Organ-on-a-Chip. Microsyst. Nanoeng..

[2] Wang B., Sanli A., Lai Z., Fu J., Adeel M., Zhu X., Yu M., Qu L., Li A., Yang Z. (2026). Next-Generation Biosensing for In Situ Monitoring. Nat. Sens..

[3] Zhou Y., Feng T., Li Y., Ao X., Liang S., Yang X., Wang L., Xu X., Zhang W. (2025). Recent Advances in Enhancing the Sensitivity of Biosensors Based on Field Effect Transistors. Adv. Electron. Mater..

[4] Zhang X., Jing Q., Ao S., Schneider G.F., Kireev D., Zhang Z., Fu W. (2020). Ultrasensitive Field-Effect Biosensors Enabled by the Unique Electronic Properties of Graphene. Small.

[5] Janićijević Ž., Baraban L. (2025). Integration Strategies and Formats in Field-Effect Transistor Chemo- and Biosensors: A Critical Review. ACS Sens..

[6] Khadem A.H., Diaz C.B., Lou L. (2025). Insights into Graphene Nanostructures, Fabrication Techniques, Mechanical, and Functional Behavior Characterization. Small Sci..

[7] Domaretskiy D., Wu Z., Nguyen V.H., Hayward N., Babich I., Li X., Nguyen E., Barrier J., Indykiewicz K., Wang W. (2025). Proximity Screening Greatly Enhances Electronic Quality of Graphene. Nature.

[8] Lasocka I., Szulc-Dąbrowska L., Skibniewski M., Skibniewska E., Strupinski W., Pasternak I., Kmieć H., Kowalczyk P. (2018). Biocompatibility of Pristine Graphene Monolayer: Scaffold for Fibroblasts. Toxicol. Vitr..

[9] Veliev F., Briançon-Marjollet A., Bouchiat V., Delacour C. (2016). Impact of Crystalline Quality on Neuronal Affinity of Pristine Graphene. Biomaterials.

[10] Knápek A., Dallaev R., Burda D., Sobola D., Allaham M.M., Horáček M., Kaspar P., Matějka M., Mousa M.S. (2020). Field Emission Properties of Polymer Graphite Tips Prepared by Membrane Electrochemical Etching. Nanomaterials.

[11] Igari T., Tsuruta R., Nishiyama Y., Adachi M., Myojin T., Asanagi K., Sasaki M., Kobayashi N., Yamada Y. (2025). Field Emission from Vertically Aligned Graphene Edges at the Apex of the Pencil Lead. Sci. Rep..

[12] Sun M., Wang S., Liang Y., Wang C., Zhang Y., Liu H., Zhang Y., Han L. (2024). Flexible Graphene Field-Effect Transistors and Their Application in Flexible Biomedical Sensing. Nano-Micro Lett..

[13] Novoselov K.S., Geim A.K., Morozov S.V., Jiang D., Zhang Y., Dubonos S.V., Grigorieva I.V., Firsov A.A. (2004). Electric Field Effect in Atomically Thin Carbon Films. Science.

[14] Szunerits S., Rodrigues T., Bagale R., Happy H., Boukherroub R., Knoll W. (2024). Graphene-Based Field-Effect Transistors for Biosensing: Where Is the Field Heading to?. Anal. Bioanal. Chem..

[15] Schedin F., Geim A.K., Morozov S.V., Hill E.W., Blake P., Katsnelson M.I., Novoselov K.S. (2007). Detection of Individual Gas Molecules Adsorbed on Graphene. Nat. Mater..

[16] Solís-Fernández P., Okada S., Sato T., Tsuji M., Ago H. (2016). Gate-Tunable Dirac Point of Molecular Doped Graphene. ACS Nano.

[17] Sun M., Zhang C., Lu S., Mahmood S., Wang J. (2024). Recent Advances in Graphene Field-Effect Transistor toward Biological Detection. Adv. Funct. Mater..

[18] Nagpal D., Singh A., Link J., Mehta A.S., Kumar A., Budhraja V. (2026). Recent Advances in Graphene-Based Field-Effect Transistor Biosensors for Disease Biomarker Detection and Clinical Prospects. Biosensors.

[19] Geiwitz M., Page O.R., Nichols M.E., Marello T., Hoar C., Akinwande D., Meyer M.M., Burch K.S. (2026). Electrical Readout Strategies of GFET Biosensors for Real-World Requirements. Biosens. Bioelectron..

[20] Ping J., Xi J., Saven J.G., Liu R., Johnson A.T.C. (2017). Quantifying the Effect of Ionic Screening with Protein-Decorated Graphene Transistors. Biosens. Bioelectron..

[21] Wang H., Qiu M., Wang C., Zhang L., Fan N., Chen Z., Liu Y., Li T., Wang Z., Zhu Y. (2025). Light-Triggered Graphene/Black Phosphorus Heterostructure FET Platform for Ultrasensitive Detection of Alzheimer’s Disease Biomarkers at the Zeptomole Level. Research.

[22] Barlian A.A., Park W.-T., Mallon J.R., Rastegar A.J., Pruitt B.L. (2009). Review: Semiconductor Piezoresistance for Microsystems. Proc. IEEE.

[23] Ni Z.H., Yu T., Lu Y.H., Wang Y.Y., Feng Y.P., Shen Z.X. (2009). Uniaxial Strain on Graphene: Raman Spectroscopy Study and Band-Gap Opening. ACS Nano.

[24] Shah R., Mohiuddin T.M. (2010). Charge Carrier Mobility Degradation in Graphene Sheet Under Induced Strain. arXiv.

[25] Irani F.S., Shafaghi A.H., Tasdelen M.C., Delipinar T., Kaya C.E., Yapici G.G., Yapici M.K. (2022). Graphene as a Piezoresistive Material in Strain Sensing Applications. Micromachines.

[26] Sahatiya P., Badhulika S. (2018). Wireless, Smart, Human Motion Monitoring Using Solution Processed Fabrication of Graphene–MoS_2_ Transistors on Paper. Adv. Electron. Mater..

[27] Park J.B., Song M.S., Ghosh R., Saroj R.K., Hwang Y. (2021). Highly Sensitive and Flexible Pressure Sensors Using Position- and Dimension-Controlled ZnO Nanotube Arrays Grown on Graphene Films. NPG Asia Mater..

[28] Blaschke B.M., Lottner M., Drieschner S., Calia A.B., Stoiber K., Rousseau L., Lissourges G., Garrido J.A. (2016). Flexible Graphene Transistors for Recording Cell Action Potentials. 2D Mater..

[29] Hodgkin A.L., Huxley A.F. (1990). A Quantitative Description of Membrane Current and Its Application to Conduction and Excitation in Nerve. Bull. Math. Biol..

[30] Wei W., Wang X. (2021). Graphene-Based Electrode Materials for Neural Activity Detection. Materials.

[31] Terranova F., Viola F.A., Di Lisa D., Massobrio P., Martinoia S., Bonfiglio A., Spanu A. (2025). Organic Charge-Modulated Transistor for Electrophysiological Measurements of Human-Derived Neurospheroids. Front. Bioeng. Biotechnol..

[32] Gu Y., Wang C., Kim N., Zhang J., Wang T.M., Stowe J., Nasiri R., Li J., Zhang D., Yang A. (2022). Three-Dimensional Transistor Arrays for Intra- and Inter-Cellular Recording. Nat. Nanotechnol..

[33] Wang Z., Tonderys D., Leggett S.E., Williams E.K., Kiani M.T., Spitz Steinberg R., Qiu Y., Wong I.Y., Hurt R.H. (2016). Wrinkled, Wavelength-Tunable Graphene-Based Surface Topographies for Directing Cell Alignment and Morphology. Carbon.

[34] Wen X., Garland C.W., Hwa T., Kardar M., Kokufuta E., Li Y., Orkisz M., Tanaka T. (1992). Crumpled and Collapsed Conformation in Graphite Oxide Membranes. Nature.

[35] Ding Y., Li C., Tian M., Wang J., Wang Z., Lin X., Liu G., Cui W., Qi X., Li S. (2023). Overcoming Debye Length Limitations: Three-Dimensional Wrinkled Graphene Field-Effect Transistor for Ultra-Sensitive Adenosine Triphosphate Detection. Front. Phys..

[36] Mukherjee P., Bahadursha N., Sultana N., Dash U.R., Chikkala P., Kanungo S., Sengupta S., RoyChaudhuri C. (2026). A Scalable Electrochemical FET Immunosensor with Crumpled Graphene for Sensitive Detection of Prostate Cancer Derived Exosomes. Nanoscale.

[37] Luo W., Guo H., Zhu X., Tian J., Wei Z., Liu M., Wang C., Sun H., Jia Y. (2025). Ultra-Thin Flexible Solid-Gated Graphene Field-Effect Transistors Fabricated Using Laser Lift-Off. Nanoscale.

[38] Kim M., Mackenzie D.M.A., Kim W., Isakov K., Lipsanen H. (2021). All-Parylene Flexible Wafer-Scale Graphene Thin Film Transistor. Appl. Surf. Sci..

[39] Hsu K.-J., Micari M., Shen Y., Li S., Song S., Agrawal K.V. (2026). Tuning Pore Size in Porous Graphene Membrane for O_2_/N_2_ Separation. Adv. Mater..

[40] Koenig S.P., Wang L., Pellegrino J., Bunch J.S. (2012). Selective Molecular Sieving through Porous Graphene. Nat. Nanotech..

[41] Jang D., Bakli C., Chakraborty S., Karnik R. (2022). Molecular Self-Assembly Enables Tuning of Nanopores in Atomically Thin Graphene Membranes for Highly Selective Transport. Adv. Mater..

[42] Yi F.-S., Bi Y.-G., Zhang X.-L., Yin D., Liu Y.-F., Feng J., Sun H.-B. (2019). Highly Flexible and Mechanically Robust Ultrathin Au Grid as Electrodes for Flexible Organic Light-Emitting Devices. IEEE Trans. Nanotechnol..

[43] Li S., Yang S., Liu J., Xu F., Wu Y., Zhang Q., He Z., Shang J., Shi C., Liu Y. (2026). Hundred-Nanometer-Thick Stretchable Liquid Metal Films for Ultra-Conformal Bioelectrodes. Adv. Sci..

[44] Zhao X., Zhang Y., Chen X., Ma C., Liu C., Liu H., Diao S. (2025). 1D Silver Nanowires/2D Graphene Composite Flexible Transparent Electrodes Induced by Superwetting Transfer. Adv. Electron. Mater..

[45] Bhatt D., Panda S. (2022). Dual-gate Ion-sensitive Field-effect Transistors: A Review. Electrochem. Sci. Adv..

[46] Huang C.-H., Huang W.-T., Huang T.-T., Ciou S.-H., Kuo C.-F., Hsieh A.-H., Hsiao Y.-S., Lee Y.-J. (2021). Dual-Gate Enhancement of the Sensitivity of miRNA Detection of a Solution-Gated Field-Effect Transistor Featuring a Graphene Oxide/Graphene Layered Structure. ACS Appl. Electron. Mater..

[47] Asgharian H., Kammarchedu V., Soltan Khamsi P., Brustoloni C., Ebrahimi A. (2024). Multi-Electrode Extended Gate Field Effect Transistors Based on Laser-Induced Graphene for the Detection of Vitamin C and SARS-CoV-2. ACS Appl. Mater. Interfaces.

[48] Wang C., Wang T., Gao Y., Tao Q., Ye W., Jia Y., Zhao X., Zhang B., Zhang Z. (2024). Multiplexed Immunosensing of Cancer Biomarkers on a Split-Float-Gate Graphene Transistor Microfluidic Biochip. Lab. Chip.

[49] Wang Z., Shaygan M., Otto M., Schall D., Neumaier D. (2016). Flexible Hall Sensors Based on Graphene. Nanoscale.

[50] Kim D.-H., Lu N., Ma R., Kim Y.-S., Kim R.-H., Wang S., Wu J., Won S.M., Tao H., Islam A. (2011). Epidermal Electronics. Science.

[51] Yang C., Su H., Zhang S., Ji H., Qi Z., Wang Y., Cheng E., Zhao L., Hu N. (2025). High Strain-Insensitive Performance Stretchable Pressure Sensor Based on the Laser-Engraved Graphene with a Serpentine Nested Structure. ACS Appl. Nano Mater..

[52] Kim J., Hong J., Park K., Lee S., Hoang A.T., Pak S., Zhao H., Ji S., Yang S., Chung C.K. (2024). Injectable 2D Material-Based Sensor Array for Minimally Invasive Neural Implants. Adv. Mater..

[53] Oh J., Lee J.S., Jun J., Kim S.G., Jang J. (2017). Ultrasensitive and Selective Organic FET-Type Nonenzymatic Dopamine Sensor Based on Platinum Nanoparticles-Decorated Reduced Graphene Oxide. ACS Appl. Mater. Interfaces.

[54] Ramoso J.P., Rasekh M., Balachandran W. (2025). Graphene-Based Biosensors: Enabling the Next Generation of Diagnostic Technologies—A Review. Biosensors.

[55] Neubert T.J., Rösicke F., Hinrichs K., Nickel N.H., Rappich J. (2024). Quantum Dot Modification of Large Area Graphene Surfaces via Amide Bonding. Adv. Mater. Inter..

[56] Yap P.L., Kabiri S., Auyoong Y.L., Tran D.N.H., Losic D. (2019). Tuning the Multifunctional Surface Chemistry of Reduced Graphene Oxide via Combined Elemental Doping and Chemical Modifications. ACS Omega.

[57] Kolb H.C., Finn M.G., Sharpless K.B. (2001). Click Chemistry: Diverse Chemical Function from a Few Good Reactions. Angew. Chem. Int. Ed..

[58] Mishyn V., Rodrigues T., Leroux Y.R., Aspermair P., Happy H., Bintinger J., Kleber C., Boukherroub R., Knoll W., Szunerits S. (2021). Controlled Covalent Functionalization of a Graphene-Channel of a Field Effect Transistor as an Ideal Platform for (Bio)Sensing Applications. Nanoscale Horiz..

[59] Kaiser D., Meyerbroeker N., Purschke W., Sell S., Neumann C., Winter A., Tang Z., Hüger D., Maasch C., Bethge L. (2024). Ultrasensitive Detection of Chemokines in Clinical Samples with Graphene-Based Field-Effect Transistors (Adv. Mater. 52/2024). Adv. Mater..

[60] Wang Y.-T., Mukundan A., Karmakar R., Chen T.-H., Dhiman H., Lin F.-M., Wang H.-C. (2025). A Comprehensive Review of Graphene-Based Biosensors: Fabrication, Applications, Characterization and Future Perspectives—A Review. APL Bioeng..

[61] Zhan J., Lei Z., Zhang Y. (2022). Non-Covalent Interactions of Graphene Surface: Mechanisms and Applications. Chem.

[62] Zhu X., Cheng K., Ding Y., Liu H., Xie S., Cao Y., Yue W. (2023). Magnetically Controlled Graphene Field-Effect Transistor Biosensor for Highly Sensitive Detection of Cardiac Troponin I. Discov. Nano.

[63] Ushiba S., Nakano T., Shinagawa A., Miyakawa N., Kato T., Yofu K., Ono T., Kanai Y., Tani S., Kimura M. (2023). Biosensing with Surface-Charge-Modulated Graphene Field-Effect Transistors beyond Nonlinear Electrolytic Screening. ACS Omega.

[64] Yin T., Xu X., Huang Z., Rosa B.G., Gaboriau D.C.A., Merali N., Keshavarz M., Bin Muhammad Mustafa A.N., Song J., Alodan S. (2025). Control of Antibody Orientation on Graphene Using Porphyrin Linker Molecules for High-Performance Graphene-Based Immuno-Biosensors. J. Am. Chem. Soc..

[65] Wang Z., Dai W., Zhang Z., Wang H. (2025). Aptamer-Based Graphene Field-Effect Transistor Biosensor for Cytokine Detection in Undiluted Physiological Media for Cervical Carcinoma Diagnosis. Biosensors.

[66] Kong D., Wang X., Gu C., Guo M., Wang Y., Ai Z., Zhang S., Chen Y., Liu W., Wu Y. (2021). Direct SARS-CoV-2 Nucleic Acid Detection by Y-Shaped DNA Dual-Probe Transistor Assay. J. Am. Chem. Soc..

[67] Guo B., Wang J., Lou F., Yuan B., Chen Z., Tang C., Chen W., Yi F., Jiang J., Hu G. (2026). Graphene Field-Effect Transistor Based Multiplexed Sensing Platform for Simultaneous Detection of Multiple Alzheimer’s Disease Biomarkers. RSC Adv..

[68] Howe L., Wang Y., Ellepola K.H., Ho V.X., Dohmen R.L., Pinto M.M., Hoff W.D., Cooney M.P., Vinh N.Q. (2025). Interfacial Photogating of Graphene Field-Effect Transistor for Photosensory Biomolecular Detection. Adv. Elect. Mater..

[69] Wang M., Zhou G., Hai W., Zhang Y., Liu Y. (2025). High-Sensitivity Detection of Infectious Disease via Fe_3_O_4_@UiO-66-NH_2_ Combined with a Graphene Field-Effect Transistor. ACS Infect. Dis..

[70] Liu Y., Zhou G., Hu S., Wang M., Du C., Xing L., Hai W., Gao G. (2026). A Graphene FET Biosensing Platform Integrated with Fe_3_O_4_@SiO_2_@Ce-Zr Bimetallic MOFs for Rapid SARS-CoV-2 Detection. Bioelectrochemistry.

[71] Salehirozveh M., Bonné R., Kumar P., Abazar F., Dehghani P., Mijakovic I., Roy V.A.L. (2025). Enhanced Detection of Brain-Derived Neurotrophic Factor (BDNF) Using a Reduced Graphene Oxide Field-Effect Transistor Aptasensor. Nanoscale.

[72] Pandey M., Bhaiyya M., Rewatkar P., Zalke J.B., Narkhede N.P., Haick H. (2025). Advanced Materials for Biological Field-Effect Transistors (Bio-FETs) in Precision Healthcare and Biosensing. Adv. Healthc. Mater..

[73] Wang L., Bao L., Qiao L., Wang J., Wang Y., Fu W., Zhang X. (2025). Epitope-Imprinted Field-Effect Transistors Overcome Debye Length Limitations for Label-Free Protein Detection. Nano Lett..

[74] Long C., McAnally J.R., Shelton J.M., Mireault A.A., Bassel-Duby R., Olson E.N. (2014). Prevention of Muscular Dystrophy in Mice by CRISPR/Cas9–Mediated Editing of Germline DNA. Science.

[75] Fan H., Ye D., Gao X., Luo Y., Wang L. (2026). Nucleic Acid-Based Field-Effect Transistor Biosensors. Biosensors.

[76] Chen S., Sun Y., Xia Y., Lv K., Man B., Yang C. (2020). Donor Effect Dominated Molybdenum Disulfide/Graphene Nanostructure-Based Field-Effect Transistor for Ultrasensitive DNA Detection. Biosens. Bioelectron..

[77] Hajian R., Balderston S., Tran T., DeBoer T., Etienne J. (2019). Detection of Unamplified Target Genes via CRISPR-Cas9 Immobilized on a Graphene Field-Effect Transistor. Nat. Biomed. Eng..

[78] Balderston S., Taulbee J.J., Celaya E., Fung K., Jiao A., Smith K., Hajian R., Gasiunas G., Kutanovas S., Kim D. (2021). Discrimination of Single-Point Mutations in Unamplified Genomic DNA via Cas9 Immobilized on a Graphene Field-Effect Transistor. Nat. Biomed. Eng..

[79] Sun M., Yu Z., Wang S., Qiu J., Huang Y., Chen X., Zhang Y., Wang C., Zhang X., Liang Y. (2025). Universal Amplification-Free RNA Detection by Integrating CRISPR-Cas10 with Aptameric Graphene Field-Effect Transistor. Nano-Micro Lett..

[80] Chen J.S., Ma E., Harrington L.B., Da Costa M., Tian X., Palefsky J.M., Doudna J.A. (2018). CRISPR-Cas12a Target Binding Unleashes Indiscriminate Single-Stranded DNase Activity. Science.

[81] Li H., Yang J., Wu G., Weng Z., Song Y., Zhang Y., Vanegas J.A., Avery L., Gao Z., Sun H. (2022). Amplification-Free Detection of SARS-CoV-2 and Respiratory Syncytial Virus Using CRISPR Cas13a and Graphene Field-Effect Transistors. Angew. Chem. Int. Ed..

[82] Gootenberg J.S., Abudayyeh O.O., Lee J.W., Essletzbichler P., Dy A.J., Joung J., Verdine V., Donghia N., Daringer N.M., Freije C.A. (2017). Nucleic Acid Detection with CRISPR-Cas13a/C2c2. Science.

[83] Liu Y., Zhong L., Zhang S., Wang J., Liu Z. (2022). An Ultrasensitive and Wearable Photoelectrochemical Sensor for Unbiased and Accurate Monitoring of Sweat Glucose. Sens. Actuat. B Chem..

[84] Hasimoto L.H., Bridges M.D., Perfecto T.M., Santhiago M., Henry C.S. (2026). Stretchable Laser-Induced Graphene Electrodes for High-Performance Electrochemical Biosensing of Lactate in Sweat. ACS Sens..

[85] Deng Z., Zeng C., Wu Q., Zhang F., Zhuang P. (2026). A Suspended Graphene Field-Effect Transistor for Ultra-Sensitive and Label-Free Detection of Cancer Biomarker miR-21. Biosensors.

[86] Ku M., Kim J., Won J.E., Kang W., Park Y.G. (2020). Smart, Soft Contact Lens for Wireless Immunosensing of Cortisol. Sci. Adv..

[87] Tripathy K., Bhattacharjee M. (2025). High-Performance Flexible and Printed Graphene-Field Effect Biosensor for Ferritin Detection. Adv. Mater. Technol..

[88] Feng Z., Zhou C., Wang Y., Dong X., Zhu H., Song H., Yang R., Wu S. (2026). Graphene Field-Effect Transistor Microarray with Simple Preparation Method, Good Stability, and High Sensitivity for Bioassay. Biosens. Bioelectron..

[89] Wang Z., Hao Z., Yu S., Moraes C.G.D., Suh L.H., Zhao X., Lin Q. (2019). An Ultraflexible and Stretchable Aptameric Graphene Nanosensor for Biomarker Detection and Monitoring. Adv. Mater..

[90] Hao Z., Huang C., Zhao C., Kospan A., Wang Z., Li F., Wang H., Zhao X., Pan Y., Liu S. (2022). Ultrasensitive Graphene-Based Nanobiosensor for Rapid Detection of Hemoglobin in Undiluted Biofluids. ACS Appl. Bio Mater..

[91] Laliberte K.E., Scott P., Khan N.I., Mahmud M.S., Song E. (2022). A Wearable Graphene Transistor-Based Biosensor for Monitoring IL-6 Biomarker. Microelectron. Eng..

[92] Wang L., Jackman J.A., Ng W.B., Cho N.-J. (2016). Flexible, Graphene-Coated Biocomposite for Highly Sensitive, Real-Time Molecular Detection. Adv. Funct. Mater..

[93] Jia Y., Zhang J., Fan Q. (2021). A Disposable DNA Methylation Sensor Based on the Printable Graphene Field Effect Transistor. E3S Web Conf..

[94] Zhang Q., Hao Y., Zeng T., Shu W., Xue P., Li Y., Huang C., Ouyang L., Zou X., Zhao Z. (2024). Modular Fabrication of Microfluidic Graphene FET for Nucleic Acids Biosensing. Adv. Sci..

[95] Zhi Y., Jian J., Qiao Y., Tian Y., Yang Y., Ren T.-L. An Ultrathin Flexible Affinity-Based Graphene Field-Effect Transistor for Glucose Monitoring. Proceedings of the 2020 21st International Conference on Electronic Packaging Technology (ICEPT).

[96] Fenoy G.E., Marmisollé W.A., Knoll W., Azzaroni O. (2022). Highly Sensitive Urine Glucose Detection with Graphene Field-Effect Transistors Functionalized with Electropolymerized Nanofilms. Sens. Diagn..

[97] Wang F., Zhang Q., Yang M., Yin B., Wong S.H.D. (2024). Recent advances in optical techniques for dynamically probing cellular mechanobiology. Biomed. Eng. Commun..

[98] Wu Y., Guo Y., Li W., Kong K., Jiang N. (2025). Matrix Swelling-Induced Precracking in Graphene Woven Fabric for Ultrasensitive Strain Sensors. ACS Omega.

[99] Paul A., Yogeswaran N., Dahiya R. (2022). Ultra-Flexible Biodegradable Pressure Sensitive Field Effect Transistors for Hands-Free Control of Robot Movements. Adv. Intell. Syst..

[100] Gao H., Wang Z., Yang F., Wang X., Wang S., Zhang Q., Liu X., Sun Y., Kong J., Yao J. (2024). Graphene-Integrated Mesh Electronics with Converged Multifunctionality for Tracking Multimodal Excitation-Contraction Dynamics in Cardiac Microtissues. Nat. Commun..

[101] Bonaccini Calia A., Masvidal-Codina E., Smith T.M., Schäfer N., Rathore D., Rodríguez-Lucas E., Illa X., De La Cruz J.M., Del Corro E., Prats-Alfonso E. (2022). Full-Bandwidth Electrophysiology of Seizures and Epileptiform Activity Enabled by Flexible Graphene Microtransistor Depth Neural Probes. Nat. Nanotechnol..

[102] Camassa A., Barbero-Castillo A., Bosch M., Dasilva M., Masvidal-Codina E., Villa R., Guimerà-Brunet A., Sanchez-Vives M.V. (2024). Chronic Full-Band Recordings with Graphene Microtransistors as Neural Interfaces for Discrimination of Brain States. Nanoscale Horiz..

[103] Ren S., He K., Cui C., Fan H., Peng H., Jiao K., Wang L. (2026). DNA Nanostructure-Assembled Metallic Nanoparticles for Biosensing Applications. Molecules.

[104] Nakatsuka N., Yang K.-A., Abendroth J.M., Cheung K.M., Xu X., Yang H., Zhao C., Zhu B., Rim Y.S., Yang Y. (2018). Aptamer–Field-Effect Transistors Overcome Debye Length Limitations for Small-Molecule Sensing. Science.

[105] Flynn C.D., Chang D., Mahmud A., Yousefi H., Das J., Riordan K.T., Sargent E.H., Kelley S.O. (2023). Biomolecular Sensors for Advanced Physiological Monitoring. Nat. Rev. Bioeng..

[106] Smith A.A., Li R., Tse Z.T.H. (2023). Reshaping Healthcare with Wearable Biosensors. Sci. Rep..

[107] Zhang R., Hao T., Hu S., Wang K., Ren S., Tian Z., Jia Y. (2022). Electrolyte-Gated Graphene Field Effect Transistor-Based Ca^2+^ Detection Aided by Machine Learning. Sensors.

[108] Kumar N., Srivastava R. (2025). Artificial Intelligence in Digital Health: Opportunities, Challenges, and Future. Biomed. Eng. Commun..

[109] Hussain S., Ghaffar M., Babar R. (2026). Overcoming Temporal and Sequential Data Challenges in Electroencephalography for Harmful Brain Activity Classification. Biomed. Eng. Commun..

